# Next steps after airing disagreement on a scientific issue with policy implications: a meta-analysis, multi-lab replication and adversarial collaboration

**DOI:** 10.1186/s12915-023-01567-5

**Published:** 2023-05-23

**Authors:** Shinichi Nakagawa, Malgorzata Lagisz

**Affiliations:** 1 Evolution & Ecology Research Centre and School of Biological, Earth and Environmental Sciences, University of New South Wales, Sydney, NSW 2052 Australia; 2 Theoretical Sciences Visiting Program, Okinawa Institute of Science and Technology Graduate University, Onna, 904-0495 Japan

**Keywords:** Replication crisis, Null hypothesis significance testing, Statistics, Team science, Open science, Reproducibility

## Abstract

Canadian policymakers are interested in determining whether farmed Atlantic salmon, frequently infected with *Piscine orthoreovirus* (PRV), may threaten wild salmon populations in the Pacific Northwest. A relevant work has been published in BMC Biology by Polinksi and colleagues, but their conclusion that PRV has a negligible impact on the energy expenditure and respiratory performance of sockeye salmon is disputed by Mordecai and colleagues, whose re-analysis is presented in a correspondence article. So, what is the true effect and what should follow this unresolved dispute? We suggest a ‘registered multi-lab replication with adversaries’.

## Absence of evidence as evidence of absence

*“Let’s be clear about what must stop: we should never conclude there is ‘no difference’ or ‘no association’ just because a P value is larger than a threshold such as 0.05”*

This quote comes from a *Nature* commentary entitled “Scientists rise up against statistical significance” [1]. Published in 2019, this commentary was widely read and shared. Therefore, we hope scientists, and even many members of the public, are aware that failing to demonstrate a statistically significant effect does not mean there is ‘no effect’. Yet, black-or-white null-hypothesis significance testing with an arbitrary *P*-value cut-off remains a standard way to report scientific findings, as pointed out again in a 2022 article calling for a more careful use of the “language of evidence” [2]. In their *BMC Biology* paper [3], Polinski and colleagues put forward three hypotheses, consistent with the concept of life-history trade-offs and the view that antiviral responses would incur measurable metabolic and other related costs to hosts. They tested these (alternative, not null) hypotheses in a laboratory experiment that assessed multiple physiological parameters during the responses to two different viruses in a species of wild salmon and rejected all three of them, based on statistically non-significant results [2]. More specifically, and of relevance to the contentious issue of whether PRV poses a threat to wild salmon populations in the Pacific Northwest, they concluded that *Piscine orthoreovirus* (PRV) infection has little or no impact on sockeye salmon. In other words, Polinski and colleagues seemed to arrive at a conclusion of ‘negligible or null effects’, mainly based on statistically non-significant results.

We point out that such practice is prevalent in the scientific literature. Indeed, a survey suggested almost half of the published papers interpret their statistically non-significant results as ‘evidence of absence’ [1]. We confess we have committed such an error as well. Then, why do so many scientists, including us, trip up on this same problem repeatedly? We believe that there are two major reasons. The first is that we cannot quite accept the fact that an expensive study is ‘inconclusive’. The second, which may be more relevant, is that to reach our ‘no effect’ conclusion, we have adopted a holistic viewpoint that does not rely solely on arbitrary *p*-value cut-offs but has incorporated other information. For example, one’s understanding of the biological system may allow for estimating a biologically meaningful effect size with high precision, the study may have high statistical power, and the conclusion may be congruent with those derived from related previous work. If the second scenario is true for Polinski and colleagues, they should still be very cautious about their conclusion. It is important to remember that supporting the null hypothesis (null effects) usually requires multiple lines of evidence other than a non-significant statistical result from a single experiment.

Mordecai and colleagues, in their correspondence [4], describe the original conclusion as ‘strident’. By re-analyzing the original data, they identified several problems with Polinski and colleagues’ study. Among these, Mordecai and colleagues showed that standard metabolic rate (SMR), an integral part of physiology, was negatively affected due to PRV. They argued that such an effect, even if small, could result in a more detrimental effect in the challenging and competitive wild environment, which was not considered in the original work. Mordecai and colleagues also indicated that low statistical power in the original design makes it likely that the original conclusion is a false negative (an effect exists but was not detected). Their conservative estimate of the statistical power of Polinski’s study included corrections for multiple testing, although experts disagree on whether these are always appropriate [5]. But even without these, the statistical power of the study to detect time-specific differences between control vs. viral-infected fish was low. Polinski and colleagues concede this point in their reply but continue to defend their much higher a priori power estimate of the ability of the study to detect a main treatment effect and their conclusion that there is little impact of viral infection. We find the criticism of low statistical power to be a serious challenge to this conclusion, along with other issues raised. Indeed, if Polinski and colleagues had demonstrated more consistent zero or near zero effects with a larger experiment or repeated experiments with their results also consistent with earlier results from other related species, we would be more convinced.

Taken together, we are more in agreement with Mordecai and colleagues, who stated that “A more objective treatment of the data makes clear that a reasonable reader could draw different conclusions than those offered in the original paper—or that it is not possible to draw firm conclusions at all” [4]. However, we should not disregard Polinski and colleagues’ work merely as a Type II error (false negative). Their paper reports the first comprehensive examination of fish transcriptional, metabolic, and histopathological parameters in response to viruses and, as they say, their integrated measurements might have been more sensitive and accurate than previous work [6]. Although inconclusive, the data remain relevant to a question that has important implications for policymakers and economic ramifications on salmon farms that act as PRV reservoirs for wild sockeye salmon. Conservation conflicts with economy or vice versa. So, what should be the next step?

## Potential next steps: research synthesis and registered replication with adversaries

We do not think that arguing the original results on statistical and other scientific bases would settle the matter (although conducting ‘equivalent tests’ would have been useful [7]). We need more evidence. There are two major pathways: research synthesis and replication. By reading the correspondence between Polinski et al. and Mordecai et al., there seem to be enough relevant studies where one can conduct an informative research synthesis, which can take a form of a systematic map or a meta-analysis (more precisely, a systematic review with meta-analysis). A systematic map would include a broad collection of relevant studies on the impact of PRV on salmon species and other fish, summarizing what has been done so far, while a meta-analysis can focus on quantitatively summarizing the negative impact of PRV on a range of physiological parameters using, say, phylogenetically controlled meta-analytic models that can aggregate evidence from multiple species.

**Fig. 1:**
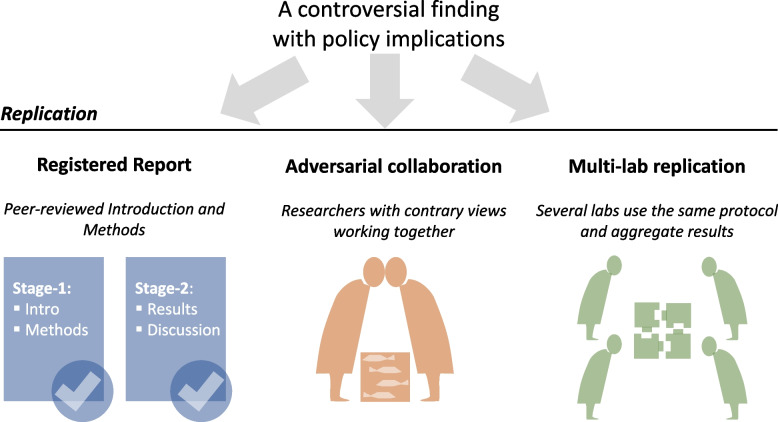
A schematic of what a “registered multi-lab replication with adversaries” would involve

However, replication is what is needed more in this instance. Here, we mean a close replication study with an improved design as required [8]. But what will such a replication study look like? We suggest it should have three properties so that replication can lead to the most robust conclusion (see Fig. 1). First, this replication should take the form of a *registered report* (*BMC Biology* is one of several biology journals which accept registered reports). A registered report has two stages. At stage 1, a proposal (i.e., the Introduction and Methods sections) is reviewed and then revised according to reviewer comments, and once accepted, the project can proceed to stage 2. At this stage, authors will conduct planned experiments and, once finished, they add their Results and Discussion sections without altering their stage 1 Introduction and Methods, although minor alternations and additions to their stage 1 plan would be acceptable [9]. This pre-commitment of hypotheses and methodology prevents questionable research practices (e.g., *p*-hacking, selective reporting and hypothesizing after results are known, or HARKing), many of which are prevalent in the field of ecology and evolution [10].

Second, this replication should include *adversarial collaboration*, where people with opposing views work together on a project. The Nobel Prize winner Daniel Kahneman and colleagues have recommended the use of such collaboration to reduce the impact of scientific biases and hidden agendas [11]. The “team Polinski” and the “team Mordecai” could work together, putting their differences aside. Then, at least, they will not argue later over, for example, their study hypotheses and study design and what may constitute meaningful biological effects.

Third, it should be a *multi-lab replication*, where several labs conduct the same experiments following the same protocol, although some heterogeneities can be introduced within and between labs (e.g., different populations and strains). In responding to Mordecai’s comment on the low statistical power, Polinski and colleagues have replied, “However, as acknowledged by Mordecai et al., the potential benefits must be weighed against the increased monetary, manpower, and animal welfare costs associated with amplified sampling of a very comprehensive study of respiratory physiology and molecular mechanisms. Specifically, samples from more than 140 individuals per treatment per time point (versus 8–16 individuals tested in our study) would be necessary to fulfill the requirements proposed by Mordecai et al. to achieve a statistical power of > 80% at the post-hoc test” [6]. We agree that testing so many fish would be difficult for one lab. Indeed, it turns out it is better to conduct several moderately powered studies (e.g., 40–50% power) and meta-analytically aggregate the results of these studies than to conduct one single high-powered study (e.g., 80%). A simulation study demonstrated that when there is heterogeneity in the system (e.g., individual and population differences), one single high-power experiment can lead to a higher type I error (false positive) rate than aggregating several smaller studies with the same total sample size [12]. Therefore, we recommend that a replication study should include more than two labs (e.g., four to five labs) so it is not only more practical but also more robust.

Finally, yet importantly, who should support such a multi-lab replication project based on adversarial collaboration? Since Canadian federal and regional governments and salmon farm industries all have an interest in the question of whether PRV poses a risk to wild salmon, we propose that they all come together to support this new way of replicating a study and thereby help to settle the matter.

## Data Availability

Not applicable.
